# The influence of air masses on human mortality in the contiguous United States

**DOI:** 10.1007/s00484-024-02745-y

**Published:** 2024-08-05

**Authors:** Cameron C. Lee, Alindomar Silva, Chibuike Ibebuchi, Scott C. Sheridan

**Affiliations:** Kent State University, Department of Geography, ClimRISE Laboratory, 433 McGilvrey Hall, 325 S. Lincoln St., Kent, OH 44242 USA

**Keywords:** Synoptic Climatology, Human Mortality, Artificial Neural Networks

## Abstract

Temperature-related mortality is the leading cause of weather-related deaths in the United States. Herein, we explore the effect of air masses (AMs) – a relatively novel and holistic measure of environmental conditions – on human mortality across 61 cities in the United States. Geographic and seasonal differences in the effects of each AM on deseasonalized and detrended anomalous lagged mortality are examined using simple descriptive statistics, one-way analyses of variance, relative risks of excess mortality, and regression-based artificial neural network (ANN) models. Results show that AMs are significantly related to anomalous mortality in most US cities, and in most seasons. Of note, two of the three cool AMs (Cool and Dry-Cool) each show a strong, but delayed mortality response in all seasons, with peak mortality 2 to 4 days after they occur, with the Dry-Cool AM having nearly a 15% increased risk of excess mortality. Humid-Warm (HW) air masses are associated with increases in deaths in all seasons 0 to 1 days after they occur. In most seasons, these near-term mortality increases are offset by reduced mortality for 1–2 weeks afterwards; however, in summer, no such reduction is noted. The Warm and Dry-Warm AMs show slightly longer periods of increased mortality, albeit slightly less intensely as compared with HW, but with a similar lag structure by season. Meanwhile, the most seasonally consistent results are with transitional weather, whereby passing cold fronts are associated with a significant decrease in mortality 1 day after they occur, while warm fronts are associated with significant increases in mortality at that same lag time. Finally, ANN modeling reveals that AM-mortality relationships gleaned from a combined meta-analysis can actually lead to more skillful modeling of these relationships than models trained on some individual cities, especially in the cities where such relationships might be masked due to low average daily mortality.

**Supplementary Information:**

The online version contains supplementary material available at 10.1007/s00484-024-02745-y.

## Introduction and background

Among the deadliest weather-related phenomena in the United States are extreme temperature events, especially extreme heat (EPA 2024). The impact of temperature on human mortality is driven by various environmental and social exposures that affect how the human body perceives thermal comfort and adapts to thermal stress (Hajat et al. 2010; Kim et al. 2011; Zuo et al 2015; Sheridan and Dixon 2017). In a warming world (Allan et al. 2021), heat events, in particular, are becoming increasingly likely (e.g. Mitchell et al. 2016), and thus, the need to further our understanding of the nuances of the temperature-mortality relationship cannot be overstated.

Many studies describe the relationship between temperature and mortality as non-linear, often exhibiting a U-shaped or J-shaped curve, with minimum mortality at some climatologically moderate temperature for a location, with increased mortality at both the extreme hot and extreme cold ends of the temperature spectrum (Anderson and Bell 2009; Gasparrini et al. 2010; McMichael et al. 2008; Donaldson et al. 2003). The cold season most often corresponds to the highest total mortality in a given location, compared to summer (Rainham et al. 2005; Kassomenos et al. 2007; Laschewski and Jendritzky 2002). There is also a lagged response of mortality to extreme temperatures, with mortality effects sometimes persisting for multiple weeks after an extreme event (Anderson and Bell 2009). However, the structure of this lagged relationship itself varies. With extreme heat events, many researchers have shown how mortality initially rises sharply but then drops below average for a few days afterward (a phenomenon referred to as mortality displacement; e.g., Saha et al. 2013; Gasparrini 2014; Armstrong et al. 2017). In addition, during cold events, there is often a multi-day delay before the mortality peak, followed by a multi-week period of increased mortality (Armstrong et al. 2017). Furthermore, seasonality and geographical differences also play a significant role in this relationship. People in higher latitudes and altitudes tend to be better acclimatized to cold but more sensitive to heat, whereas the opposite holds for those in lower-latitude regions (Haines et al. 2006; Guo et al. 2016; Macintyre et al. 2021).

In addition, human thermal comfort is not a function of temperature alone, but rather the entire “multivariate situation” to which individuals are exposed, including, humidity, solar radiation, and wind (Matzarakis 2021). As such, multiple thermal indices have been developed in an effort to capture this holistic suite of meteorological components influencing thermal-related human morbidity and mortality: the heat index, wet bulb globe temperature, apparent temperature, and the Universal Thermal Climate Index, among others (Matzarakis 2021; Spangler et al. 2022). However, many of these indexes have some limitations, in particular, they often do not inherently incorporate the latitudinal/geographical and seasonal variations in the temperature-mortality relationship noted above. This is where a recently-developed air mass (AM) classification could help fill in the gap.

Air masses are one of the typical units of measure through which the synoptic-scale atmosphere is often examined (Lee and Sheridan 2015). One of the early classifications for identifying air masses was the Spatial Synoptic Classification (SSC), developed by Sheridan (2002). The SSC uses the temperature, humidity, pressure, wind speed, wind direction, and cloud cover to classify every day at a location (usually an airport weather station), into one of seven main weather types. Due to its ease of use, simple understanding, and its multivariate nature, the SSC has been used extensively in temperature-related mortality research (Hondula, et al. 2014). More recently, however, Lee (2014) introduced a Gridded Weather Typing Classification (GWTC) system based upon uniformly gridded reanalysis data that, at first, was designed to classify surface air masses across the United States. Of particular relevance to this current research, the GWTC incorporates both seasonal and geographical variability into the multivariate classification of its air masses. That is, GWTC air masses are defined relative to the climatological mean (and standard deviation) for each weather variable (the same six variables used in the SSC), at every location, and for every day of the year. In essence, this pre-packaged dataset of daily air masses thus inherently accounts for a population’s acclimatization to their city’s ‘normal’ thermal environment. In one of the early applications of the original GWTC, Lee (2016) showed how these AMs had significant impacts on wintertime cardiovascular mortality in 19 cities in the United States.

In 2020, the original GWTC was redeveloped (hereafter: the GWTC2) to expand the system to a global scale, and to be more sensitive to extreme conditions, and thus, better at partitioning out extreme AMs from the more mundane (Lee 2020). Building upon the work of Lee (2016), the goal of this research is to apply these GWTC2 AMs to investigate how lagged mortality rates vary after a population’s exposure to these updated AMs, while noting the spatial and seasonal variability to these relationships.

## Data and methodology

### Data

Mortality data were obtained from the National Center for Health Statistics in the United States, spanning the years 1975 to 2018. The analysis focuses on all-cause mortality across all age groups in the 61 largest metropolitan statistical areas in the country, with geographic boundaries defined by the 2010 United States Census (see list of cities in Supplementary Material). Days which had mortality totals greater than 4 standard deviations above expected levels were individually examined. Those days for which increased mortality could be attributable to factors other than extreme temperature, such as transportation accidents, terrorism, tornadoes, fires, and hurricanes were excluded.

Separately for each city, raw daily mortality counts were transformed into deseasonalized and linearly-detrended standardized anomalies (i.e., z-scores; as described by Lee et al. 2024). First the raw data are linearly detrended using least-squares regression. The resulting detrended data are then deseasonalized using a multi-step process (see supplementary material, Figure S9). Firstly, seasonal curves (baselines) of average/mean mortality and seasonal curves (baselines) of standard deviations of mortality must be developed. To do this, the 12 monthly means and the 12 monthly standard deviations are calculated. Then these month-to-month statistics are smoothed and interpolated into day-of-year means and day-of-year standard deviations by fitting a piecewise cubic spline, centered on the middle day of each month (i.e., the middle day of each month is equal to that month’s mean (or to that month’s standard deviation, when calculating that statistic)). These smoothed curves therefore represent the detrended day-of-year/seasonal cycle of mean mortality and of standard deviation of mortality, for the entire 44-year time series. The final step is then to use the common z-score equation, whereby for each day in the time series, we subtract the detrended day-of-year mean mortality from the detrended raw mortality, and then divide the difference by the detrended day-of-year standard deviation of mortality. The resulting timeseries, therefore, quantifies the detrended and deseasonalized standardized anomalous mortality for each city (referred to as Zmort from here on).

**Fig. 1:**
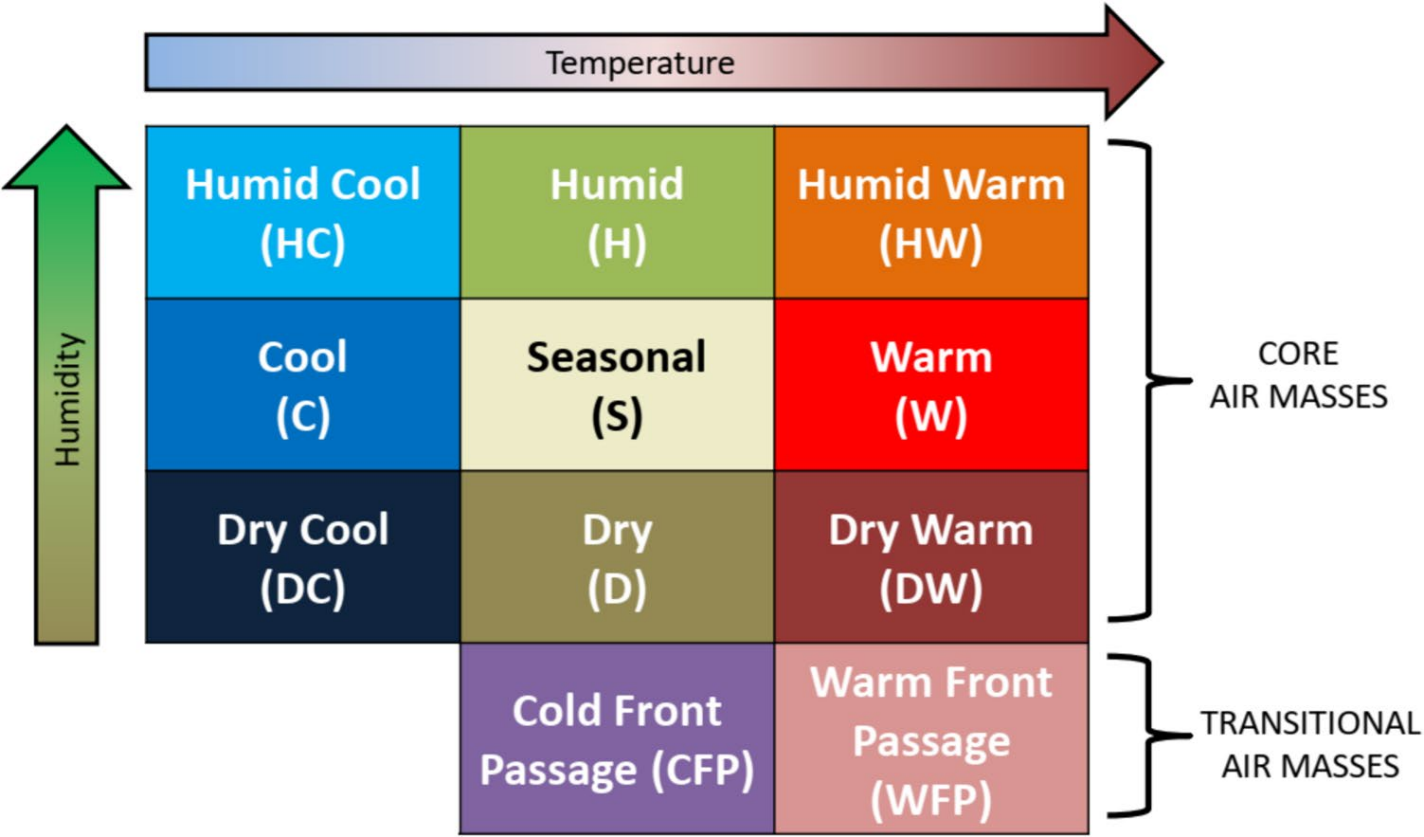
GWTC2 Air masses (modified from Lee 2016)

Air mass (AM) data were obtained from version 2 of the gridded weather typing classification (GWTC2; available here: https://www.personal.kent.edu/~cclee/gwtc2global.html). Underlying the classification of air masses in the GWTC2 are 2-m temperature, 2-m dew point, 10-m u-wind and v-wind components, total column cloud cover, and sea-level pressure from the Climate Forecast System (CFS) reanalysis (CFSR, from 1979-March 2011) and the CFS operational analysis (CFS-OA; from April 2011 through 2018) multiple times per day. As first described in Lee (2014) and updated in Lee (2020), through a multi-step process, these data are clustered to categorize each day at each location (at 0.5° × 0.5° spatial resolution) into one of 11 AMs – including nine core AMs and two transitional AMs (Fig. 1). Importantly, these air masses are “seasonally and geographically relative,” meaning that they identify multivariate weather conditions based upon how anomalous the conditions are compared to averages (and variability) for the exact day of the year and the exact location. For each of the 61 cities, the GWTC2 AM data from the nearest land-based gridpoint to each city’s main airport was chosen to use in the analysis below, from 1979–2018 – the 40-year (14,610-day) overlapping period with the mortality data described above.

### Methods

Summary relationships between AMs and mortality were examined in three ways: 1) simple averaged Zmort response by lag day after the occurrence of an AM; 2) the relative risk (RR) of excess mortality by lag day after the occurrence of an AM; and 3) a one-way analysis of variance (ANOVA) testing the ability of the GWTC2 to partition Zmort between the various AM (i.e., is the categorical GWTC2 variable a significant predictor of Zmort). In addition, many results were subsequently stratified by meteorological season (DJF – winter; MAM – spring; JJA – summer; SON – autumn) and graphed; and stratified by city, and mapped. In addition, a meta-analysis was also undertaken wherein the Zmort (and lagged AMs) for all 61 cities were simply concatenated into a single dataset (such that this combined dataset had 891,210 observations (14,610 days × 61 cities)) to effectively examine US-wide results. Relative risks are the ratio of the probability of excess mortality (days when Zmort > 0) occurring given a certain exposure (i.e., the occurrence of an AM x-days ago), over the probability of excess mortality occurring when that exposure does NOT occur. So, for example, if the empirical probability is that excess mortality occurs 75% of the days that the Dry-Cool (DC) AM occurs, but only occurs 50% of the days on which any other AM occurs, then RR = 1.5 (75% divided by 50%). Such a result would be interpreted as the DC air mass being associated with a 1.5x  greater risk of excess mortality compared to non-DC days. The 95% confidence interval (CI); was also computed for each of the above RR analyses to determine statistical significance of the results. Statistical significance was implied for any result where the CI did not include 1.0 (the RR baseline).

To further examine the AM-mortality relationship, including the interaction/sequencing impact of AMs on Zmort, two separate artificial neural network (ANN) modeling methodologies were employed: 1) training 61 individual ANN models, one model trained on the data for each city; and 2) training a single ANN model on the combined data of all 61 cities. For both modeling methods the predictor variables were the dummy-variables for AMs over the 0 to 4-day lag period, and two sinusoidal seasonal curves (one curve with inflections near the solstices, one curve with inflections near the equinoxes) to incorporate the differing/opposing effects of AMs on mortality according to the time of year. The predictands in all models were Zmort over a 20-day period, from 0 to 19 days of lead time after the occurrence of an AM. Identical modeling architectures were used for both methodologies: single-layer, 10-neuron, cascade-forward shallow ANNs were trained on an interleaved 75% of the dataset, with 25% held out for validation. The Levenberg–Marquardt (Hagan and Menhaj 1994) method of optimization was used, along with an internal early-stopping technique employed to mitigate model overfitting (Prechelt 1998). For visualization in the results below, the outputs from these models (i.e., modeled Zmort) were stratified by AM, and averaged by day-of-the-year (1–365) and lag day (0–19), resulting in a 365 × 20 matrix. Finally, this 365 × 20 matrix for the Seasonal AM was then subtracted from that of each of the other 10 air masses, highlighting the modeled AM-mortality differences from seasonal weather, for each AM.

## Results and discussion

### AM-mortality relationships

The results from the one-way ANOVA reveal that AMs are statistically significant predictors of lagged mortality in most cities (Table 1). Indeed, every city has at least one lag-day (of the 20) on which a statistically significant association exists between AMs and mortality in every season. Winter exhibits the most significant relationships, especially at extended lag times, peaking 2–4 days after an AM occurs – a pattern that also manifests in autumn, and to a lesser extent, in spring. In summer, the significant results are largely confined to the 0–1 days after an AM occurs, after which time, few cities exhibit significant relationships between AMs and mortality. This overall seasonality in the lagged structure of mortality responses to temperature extremes is fairly well-known (e.g., Sheridan and Kalkstein 2010).

**Table 1:** Number of cities (out of 61) with statistically significant results from a one-way ANOVA (testing average standardized anomalous mortality (Zmort) by the categorical GWTC2 air mass variable), by lag day and season

Lag Day	Winter	Spring	Summer	Autumn
0	28	40	48	31
1	41	26	40	25
2	53	38	20	43
3	49	40	18	45
4	46	37	12	40
5	39	32	13	36
6	37	27	10	35
7	38	27	8	26
8	41	24	5	19
9	30	22	9	15
10	33	22	9	19
11	24	22	7	12
12	26	15	13	8
13	22	18	9	6
14	18	13	10	9
15	21	10	9	5
16	21	13	9	8
17	20	12	11	3
18	20	13	8	4
19	16	7	7	9

**Fig. 2:**
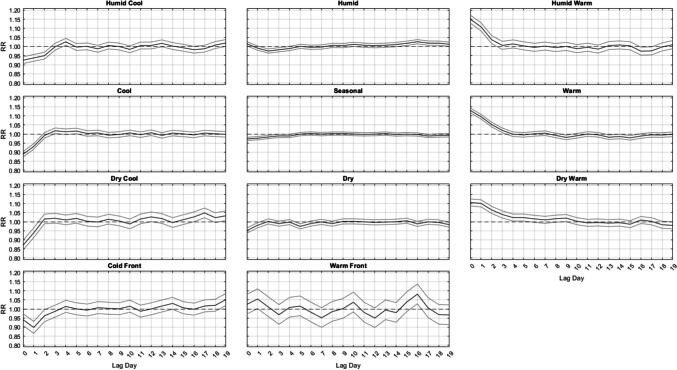
Domain-wide combined relative risk (y-axis) of excess mortality in summer, by lag day (x-axis) for each air mass (black line), along with 95% confidence intervals (gray lines)

In terms of the varied responses *between different* AMs, summertime lagged mortality responses are somewhat different than those in most of the other seasons when considering all 61 locations (Fig. 2). Most notably, as implied in the ANOVA results, nearly all significant relationships in summer are strongest at Lag0 and do not persist past the first 3 to 4 days after an AM occurs, whether those relationships are harmful (i.e. leading to excess mortality, RR > 1.0) or protective (i.e. leading to less mortality; RR < 1.0). All three cool AMs (DC, C, and HC) have a significant protective effect at Lags 0–1. All three warm types (DW, HW, and W) result in a strong immediate increase in mortality for several days, from lags 0–2 for HW, lags 0–3 for W, and lags 0–5 for DW. Notable is that after this initial increase, the RR of mortality stays very near statistical unity (RR = 1.0) for nearly the entire two-week time period after the occurrence of these AMs, with only a handful of days being either significantly higher or lower than RR = 1.0.

**Fig. 3:**
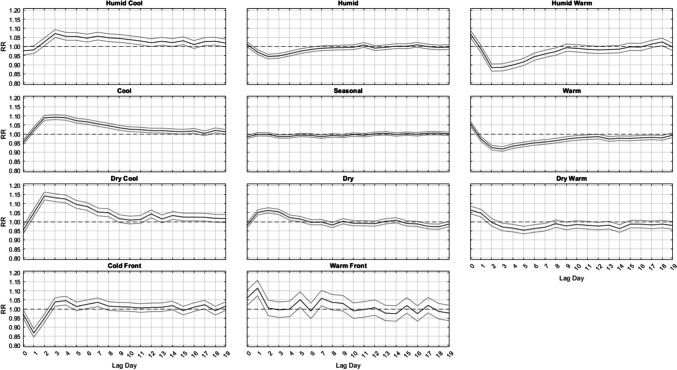
Same as Fig. 2, except for Autumn

**Fig. 4:**
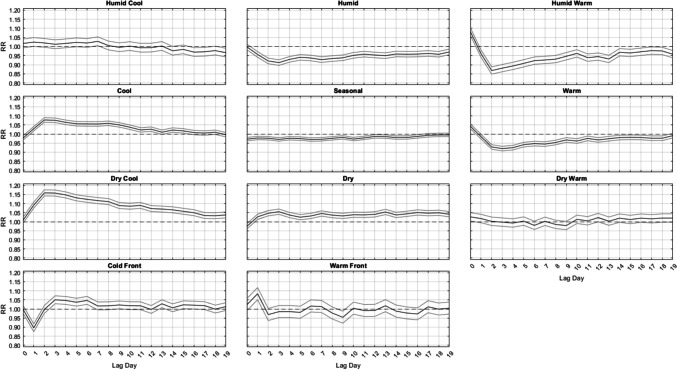
Same as Fig. 2, except for Winter

**Fig. 5:**
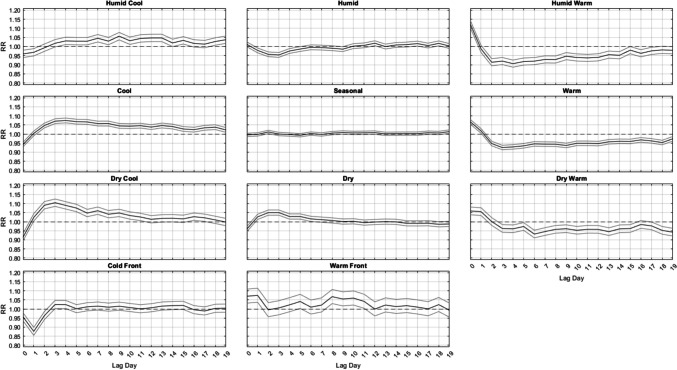
Same as Fig. 2, except for Spring

Outside of summer, the results are much more similar across seasons (Figs. 3, 4 and 5). That is, from autumn into winter and through spring, it is largely the DC and C air masses that are followed by increased mortality, especially in winter, about 2–3 days delayed after their occurrence, where relative risks approach RR = 1.16 (95% CI = [1.15, 1.17]) for DC and RR = 1.08 (95% CI = [1.06, 1.09]) for C, with neither exhibiting a significant effect on the day they occur. This result follows closely with results from Lee (2016), showing that DC exhibits a significant increase in cardiovascular deaths in winter. The HC air mass shows a similar delayed response, but excess mortality is only apparent in the shoulder seasons (spring and autumn) after 3 days of lag. Of note, the HW and W air masses are associated with a significant increase in mortality on the day they occur – a result that spans all seasons (but is most intense in summer). However, unlike the results for summer, on the days immediately following this initial mortality increase after HW and W occur, mortality then becomes significantly lower than average. This mortality reduction is most pronounced in HW and W, while the DW air mass exhibits this effect more mildly, and mostly in spring and autumn.

While the aforementioned relationships vary in *magnitude* on a season-by-season basis, the *general shape* of the lag-curves for each AM is fairly steady across the seasons (Figs. 2, 3, 4 and 5). For example, when the DC air mass occurs, there is a general rise at Lag0 to Lag2, a plateau from Lag2 to Lag4, and then a very gradual decline for the next 5 days. In the winter, this bump-like curve starts at RR = 1.00, increases to RR = 1.16 at Lag2, and then declines to RR = 1.04 over the next 17 days. However, even in summer, this bump-like *shape* for DC persists, but at different *magnitudes*; instead starting at RR = 0.87 at Lag0, increasing to RR = 1.02 at Lag2, and then decreasing to RR = 0.95 to RR = 1.00 over the next several days.

One of the more consistent results across all seasons is the protective effect offered by the passage of a cold front (CFP), which yields significantly decreased mortality one day after it occurs, ranging from RR = 0.88 to RR = 0.90 in every season. CFP would typically be followed by either a DC or C air mass one day later, both of which are associated with decreased mortality on the day they occur (Lag0) in most seasons. However, in winter, the C air mass is the only one of the three cool AMs that yields a significant RR < 0.97 at Lag0, suggesting that the significance of the CFP Lag1 result may be somewhat independent from the effects of the cool AMs that come after it. The other transitional air mass – WFP – also shows a seasonally-consistent significant effect on mortality one day after it occurs, however it is a significant *increase*. This supports prior research that suggests sudden changes in weather conditions can impact mortality (Allen and Lee 2014; Coleman 2005).

**Fig. 6:**
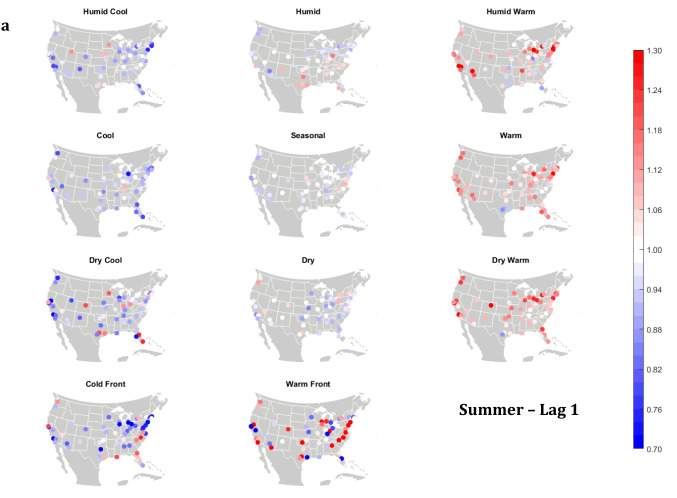

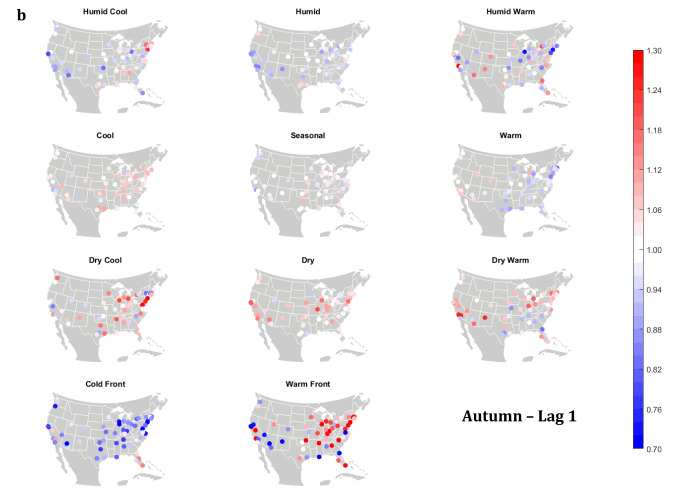

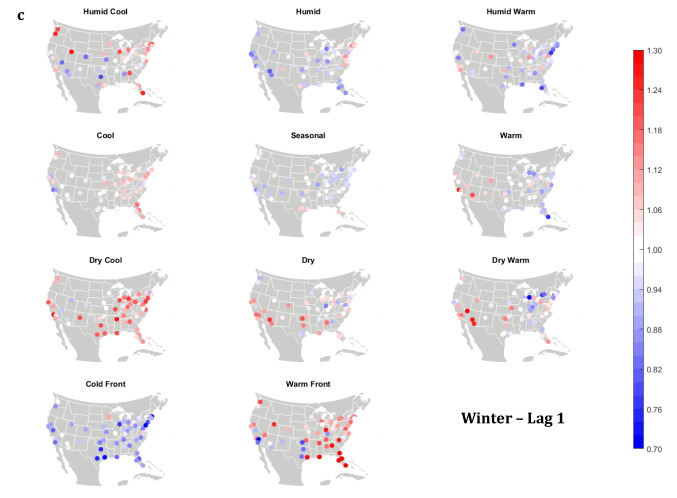

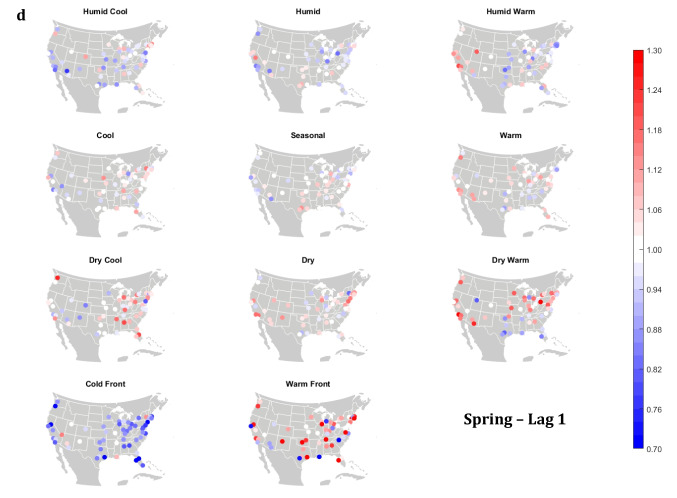
City-by-city relative risk of excess mortality at Lag1 for each air mass in **a** summer; **b** autumn; **c** winter; **d** spring

**Fig. 7:**
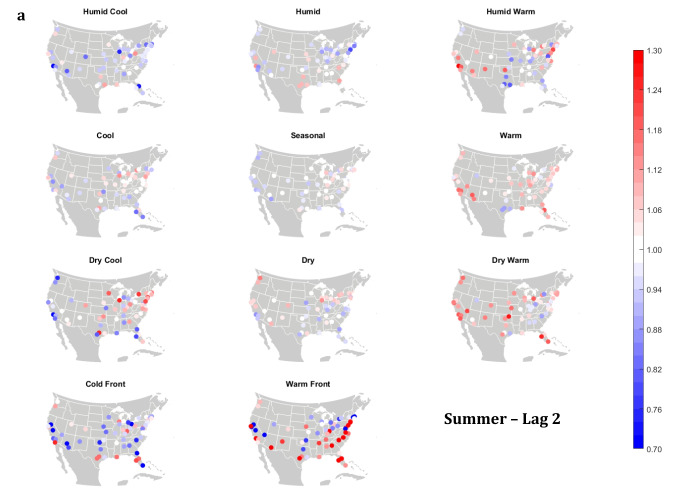

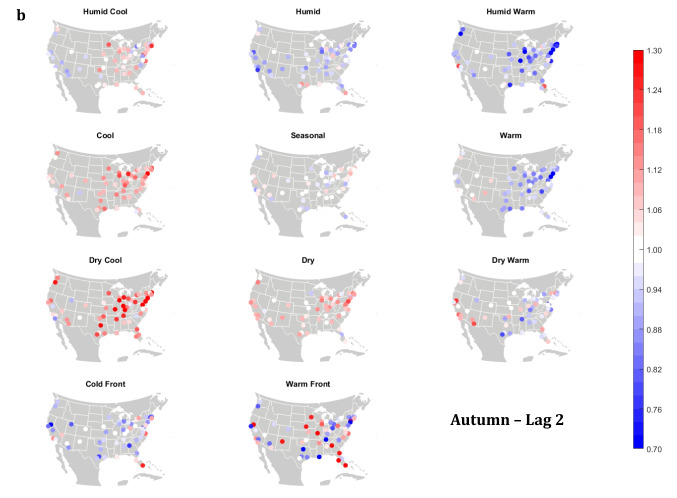

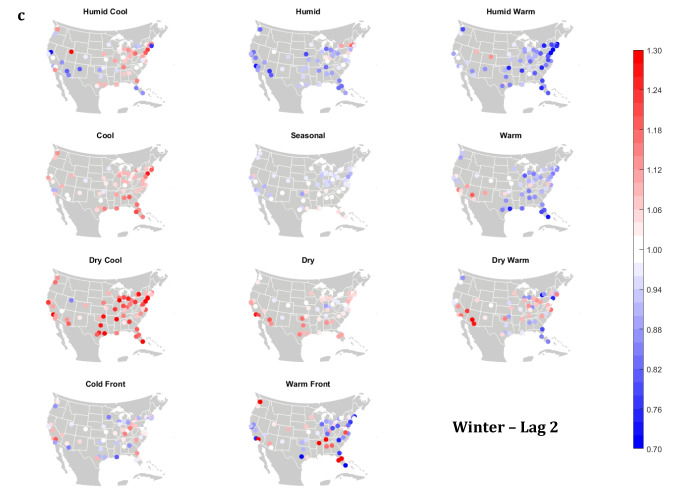

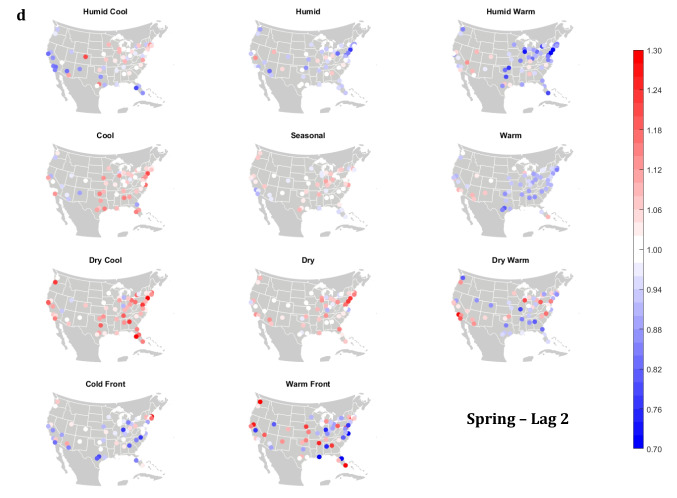
Same as Fig. 6, except for Lag2 in **a** summer; **b** autumn; **c** winter; **d** spring

The domain-wide results are fairly consistent on a city-by-city basis (Figs. 6 and 7). This said, some spatial variability does exist. For example, in the (humid-subtropical) southeastern quadrant of the US, summertime HW has markedly less of an immediate (Lag0-1) impact on increasing mortality in comparison to other locations (Fig. 6), supporting the notion that high heat and humidity are less impactful on inhabitants of climates that are normally used to such conditions (Anderson and Bell 2009; Curriero et al. 2002). Specific to the Southeastern US, Sheridan and Kalkstein (2010) also noted that this region experiences decreased summertime mortality associated with traditionally oppressive air masses. Instead of HW, in this region, the W and H AMs appear to be associated with larger magnitude mortality increases in many locales, as residents here may change their behavior to avoid thermal stress during the most oppressive (HW) conditions (e.g., using air conditioning; Anderson and Bell 2011). Winter also shows some interesting spatial variability with the D and DW air masses, both of which appear to have a protective effect in the more northern and eastern locations, while they have more of a harmful effect in the more southerly latitudes and in the west, corresponding to results from Lee (2016) using the first version of the GWTC. The wintertime effect of the WFP at Lag1 also has a strong regional preference – leading to excess mortality in most locations but having a protective effect in the southwestern quadrant of the US. As noted above, prior research has highlighted that transitional atmospheric setups that mimic warm front passages have been linked specifically to cardiovascular issues, in particular in the northern and/or eastern portions of the US (Coleman 2005; Allen and Lee 2014). Outside of summer, the Lag1 protective effect of CFP shows near spatial ubiquity (except for a few Florida cites in autumn).

Meanwhile, the 2-day lagged effect of increased mortality after DC and C air masses occur is also nearly ubiquitous, along with the opposite impact for HW at this lag time in most seasons (Fig. 7). Again, summer stands out as the non-conforming season at Lag2, when the effects of HW and DC vary across the US. In summer, the transitional AMs seem to show the highest magnitude results at Lag2. Two days after the CFP AM occurs there is still a lingering protective effect across much of the US, with the exception of the extreme southern US and a handful of cities in the Midwest and northwest. Despite US-wide non-significance of the WFP results, there appears to be a sharp geographic discontinuity where WFP is associated with substantial increased mortality in the southern and coastal cities, while the central US and inland locations have markedly reduced mortality.

### Modeling results

**Fig. 8:**
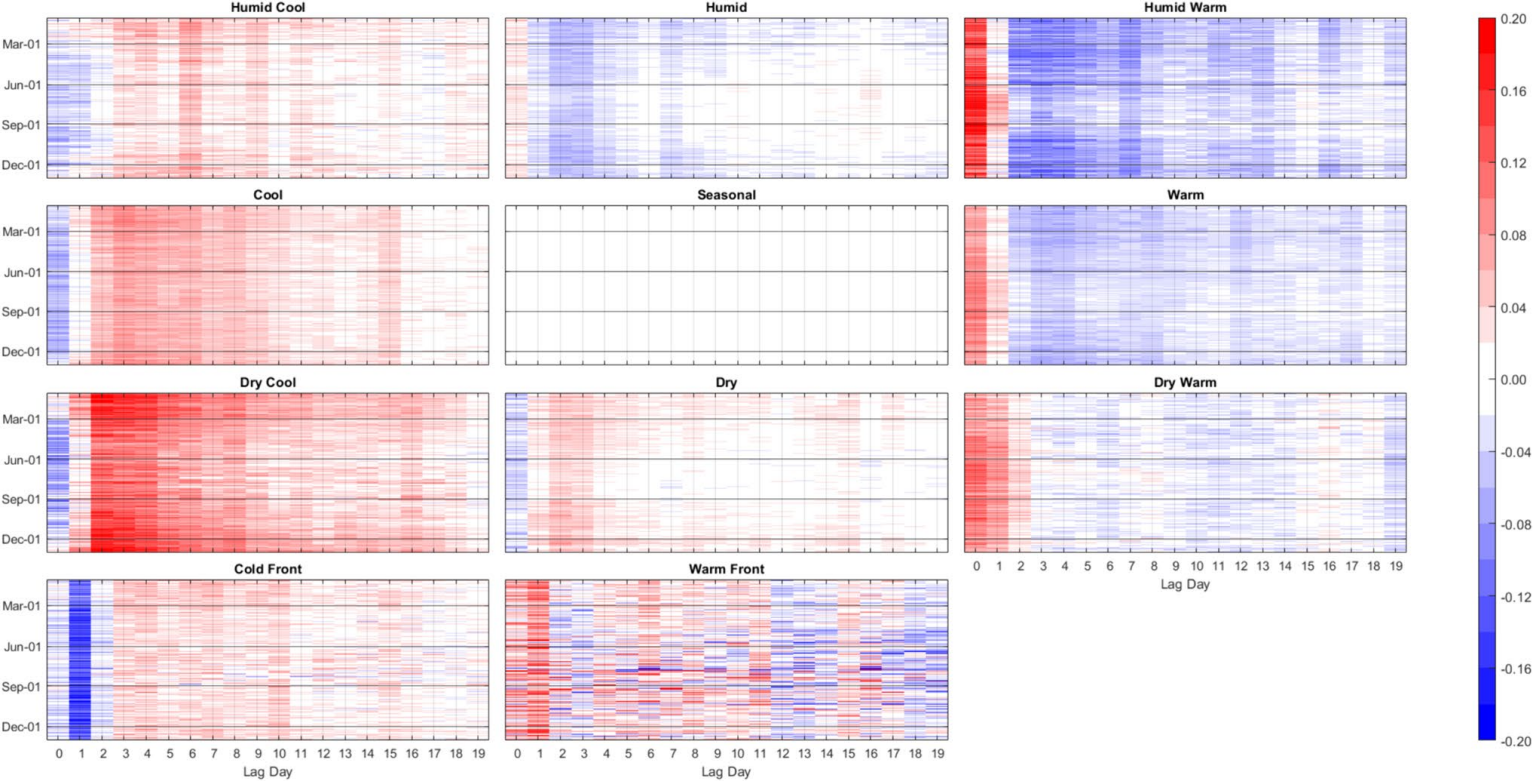
Averaged output of the 61 separate-city ANN models. Colors/units are Zmort (standardized anomalous mortality) differences between each air mass and the seasonal air mass (thus, for the seasonal AM, Zmort = 0 at all points), averaged by day of the year (y-axis) and lag day (x-axis) after the occurrence of each AM. Output is based on training and validation datasets. Higher/positive values (darker reds) indicate increased mortality relative to that of the Seasonal AM, lower/negative (darker blues) indicate decreased mortality relative to that of the Seasonal AM

**Fig. 9:**
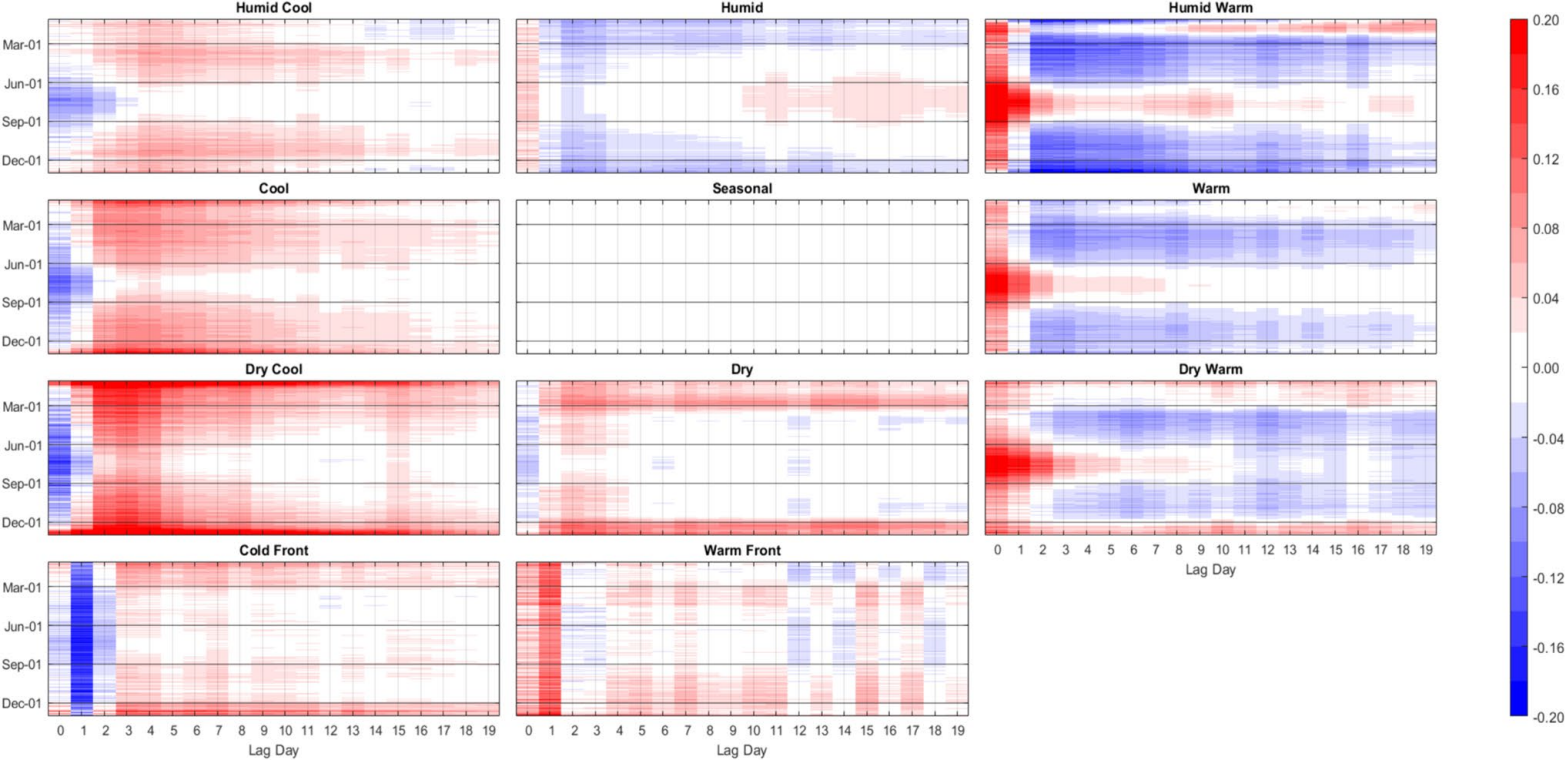
Same as Fig. 8, except using the output of the combined-meta ANN model

**Fig. 10:**
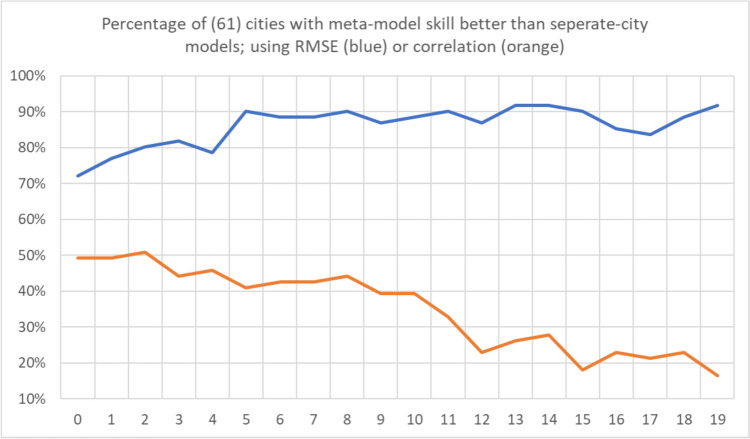
The percentage (y-axis) of 61 cities where meta-model results in that city were more skillful than results for the separate-city model for that city. Skillfulness is measured using root mean squared errors (RMSE, blue) between the model output and actual observed Zmort, and Pearson correlation (orange) between the model output and actual observed Zmort, based on both the training and validation portions of the datasets

Again, the ANN modeling results were completed two ways: 1) training 61 individual models, one on each city; and 2) training one meta-model on the combined data of all 61 cities. Unsurprisingly, the separate-cities models showed more variability in the AM-mortality relationship from city to city than the meta-model results (Figs. 8 and 9). Importantly however, this did *not* mean that the resulting city-customized models were more accurate. In terms of root mean squared error, the meta-model performed markedly better than the model customized to each city. On average, the meta-model performed superior to the separate city model in/on 86% of the cities and lag days, ranging from being superior in 72% (44 of 61) of cities on Lag0 to 92% (56 of 61) of cities on Lag19 (Fig. 10). Moreover, the cities where the meta-model performed the best relative to a city’s customized ANN model, tended to be the smaller cities, with fewer average daily deaths, and thus, generally were the more difficult cities to model individually. Indeed, we found a Pearson correlation of r = -0.67 between a city’s average daily number of deaths (a proxy for population) and the relative improvement in the RMSE of the meta model over the RMSE of a city’s individual ANN model. It should be noted, however, that compared to the RMSE results, correlation-based measure of skill showed mixed results in terms of the skill of the separate city models versus that of the meta-model, especially in the first few days after AM occurrence (Fig. 10). Nonetheless, the RMSE results are similar to Gasparrini et al. (2022), where a meta-analysis approach was more robust for areas that had weak signal between the weather and human mortality relationship. Effectively, this suggests that the ‘easier-to-model’ cities (either due to greater population or just a stronger relationship between weather and mortality) provide some predictive power in assessing key weather-mortality relationships in the other locations. Furthermore, meta-modeling may be the more effective (and simplistic) modeling strategy compared to examining dozens of separate model results, and it may yield more accurate insight into temperature/AM-mortality relationships than post-hoc aggregation of model outputs (e.g., those shown in Fig. 8). As such, the combined-meta models (i.e., those displayed in Fig. 9) are examined in more detail from here on.

The meta-model echoes many of the findings noted using the raw data above. In particular, Fig. 9 exhibits: 1) the delayed, but long-lasting harmful effect of the C and DC AMs in the winter and their protective immediate effect in all other seasons; 2) the immediate increase in mortality after any of the three warm AMs in all seasons, but most concentrated in summer; and 3) the 1-day lagged mortality response to the CFP (protective) and WFP (harmful) throughout all seasons as well. The meta-model also helps add robustness to some of the more nuanced results noted in the section above. One of these is the *lack* of an extended period of reduced mortality in summer following the initial uptick of mortality from Lag0-2 after one of the 3 warm AMs (DW, W or HW) occurs. Figure 9 shows that when one of these 3 warm AMs occur in summer – which would be at the highest *raw/absolute* temperatures – there is a significant immediate increase in mortality which gradually tapers to unity over the period of 7–10 days. Only for the DW type do we see a reduction in mortality afterwards. This figure also shows that when anomalously warm conditions occur *outside* of summer (i.e., when conditions that are hot for the season, but are somewhat mild compared to summer thermal stress for that location), the immediate increase in mortality is mostly offset by an extended period of reduced mortality (e.g., see supplementary material figures S6-S8). Of note, the averaged results of the *separate cities* ANNs (Fig. 8) do show an extended period of slightly reduced mortality after the initial uptick, though summer results (with HW especially) appear to be milder by comparison to all other seasons.

Interestingly, in winter, the DW air mass has a very different effect on mortality than its W and HW counterparts when analyzed using the meta-model. While all 3 warm AMs lead to immediate increases in wintertime mortality, this is followed by a substantial decrease in mortality in the days after HW and W. In wintertime, however, the mortality stays higher than average for nearly the entire lag-window after the DW air mass occurs. This DW result in winter is much more like the effects of the Dry AM during this season (compared to the results of the other two warm AMs), and combined, these results suggest that aridity might play a prominent role in impacting winter mortality.

## Summary and conclusions

This research is the first to apply the latest edition of the GWTC – the GWTC2 – to identify the relationships between the occurrence of multivariate air masses and human mortality. Beyond being the first to use the GWTC2 in this manner, this research also updates prior research done with the original GWTC (Lee 2016), by expanding the analysis in space (61 cities vs. 19 cities), time (mortality data through 2018 vs. 2010), seasons (all seasons vs. just winter) and cause of death (all cause vs. cardiovascular only). Results show that AMs are significantly related to anomalous human mortality in most US cities, and in most seasons. Many of these relationships mirror those outlined in prior research using the GWTC’s predecessor, the SSC, and/or a continuous measure of human thermal comfort (Hondula et al. 2014; Anderson and Bell 2009). Among these ‘anticipated results’ are that summertime heat events and wintertime cold events are related to increased mortality in the days afterwards (at 0–1 lag for heat, and Lag 2–4 for cold). Additionally, with the exception of wintertime dryness, most results indicate that humidity plays a small role (relative to that of temperature) in the lagged thermal-mortality relationship. However, the more interesting results that emerged from this research are the following:

1. Two of the three cool AMs (C and DC) each show a strong, but delayed mortality response in ALL seasons (especially outside of summer), with peak mortality 2 to 4 days after they occur, with the Dry-Cool AM having nearly a 15% risk of excess mortality on average.
2. The most *seasonally consistent* results are with transitional weather, whereby cold fronts are associated with a significant decrease in mortality 1 day after they occur, while warm fronts are associated with significant increases in mortality at that same lag time.
3. In all seasons, the HW and W air masses are associated with increases in deaths 0–1 days after they occur, and especially in summer. In most seasons, these increases in mortality are largely offset by reduced mortality at extended lag times. However, in summer, this reduction in mortality (after the initial uptick) largely disappears, as no apparent decrease in mortality is observed after the period of increased mortality.
4. The DW air mass, while lower in magnitude than HW’s impact on mortality, shows a slightly longer period of significantly increased mortality than HW after it occurs in summer.
5. Using a meta-analysis can lead to more skillful modeling of AM-mortality relationships than training models on individual cities, especially in cities where such relationships might be masked due to low average daily mortality.

This last conclusion suggests that, by leveraging data from larger, but climatologically-similar cities, the temperature-related mortality relationships in smaller towns and more-rural locations might be easier to model than often assumed, despite their often prohibitively small sample sizes of daily mortality.

Considerable research has focused on finding an ideal metric for measuring human thermal comfort in particular situations/locations/applications (e.g., Matzarakis 2021; Urban and Kyselý, 2014; Thorsson et al. 2021; Grundstein and Vanos 2021). Many multivariate metrics (e.g., apparent temperature) can be problematic for some applications because ideal thermal comfort varies by location/latitude/climate zone, meaning that the same raw apparent temperature (of say 35 °C) in two different locations may have very different mortality responses in those locations. Others (e.g., a simpler percentile threshold method) can be problematic because they simply cannot identify *seasonally-relative* extreme conditions that can be harmful. Because of this, forecasts using many of these metrics still need to be *interpreted* relative to location (or season) to give it meaning with regard to how it might impact human health. This additional burden of ‘relative interpretation’ is likely a nontrivial reason why it is difficult to find a universal metric for issuing extreme temperature advisories, watches, and warnings across endlessly varying climate regions (Hawkins et al. 2017). While AMs are not developed specifically to measure physiological environmental strain, the GWTC2 AMs are defined relative to location and season; and in this regard, the utilization of AMs for issuing watches and warnings might yield a simpler way of communicating thermal stress hazards to the general public across widely varying climate regions. As such, we hope future research will examine the efficacy – both operationally and scientifically – of using the GWTC2 air masses alongside these other well-established biometeorological indices in this pursuit.

## Supplementary Information

Below is the link to the electronic supplementary material.

Supplementary file1 (PDF 1307 KB)

## Data Availability

All GWTC-2 data are available at https://www.personal.kent.edu/~cclee/gwtc2global.html or by emailing Dr. Cameron C. Lee at cclee@kent.edu.
