# Supplementary material for: The influence of air masses on human mortality in the contiguous United States
Source: Int J Biometeorol. 2024 Aug 5;68(11):2281–96. doi: 10.1007/s00484-024-02745-y (PMC11519110; doi:10.1007/s00484-024-02745-y)
Supplement: Supplementary file 1 — Supplementary file1 (PDF 1307 KB) [file 484_2024_2745_MOESM1_ESM.pdf]

SUPPLEMENTARY MATERIAL FOR:

The Influence of Air Masses on Human Mortality in the Contiguous United States

Cameron C. Lee[1*]
Alindomar Silva[1]
Chibuike Ibebuchi[1]
Scott C. Sheridan[1]

1 – Kent State University, Department of Geography, ClimRISE Laboratory
* - Corresponding Author Information:
433 McGilvrey Hall
325 S. Lincoln St.
Kent, Ohio 44242 USA
Ph: +1 (330) 672-0360
Email: cclee@kent.edu

TABLES:

Table S1: List of cities examined in this study.

City and State	Abb.	Airport Lat	Airport Lon	GWTC Lat	GWTC Lon
Albuquerque NM	'ABQ'	35.04	-106.61	35.00	-106.50
Albany NY	'ALB'	42.75	-73.80	42.50	-74.00
Atlanta GA	'ATL'	33.64	-84.43	33.50	-84.50
Austin TX	'AUS'	30.19	-97.67	30.00	-97.50
Hartford CT	'BDL'	41.94	-72.68	42.00	-72.50
Birmingham AL	'BHM'	33.56	-86.75	33.50	-87.00
Nashville TN	'BNA'	36.12	-86.68	36.00	-86.50
Boston MA	'BOS'	42.36	-71.01	42.50	-71.00
Buffalo NY	'BUF'	42.94	-78.73	43.00	-78.50
Baltimore MD	'BWI'	39.18	-76.67	39.50	-76.50
Cleveland OH	'CLE'	41.41	-81.85	41.50	-82.00
Charlotte NC	'CLT'	35.21	-80.95	35.00	-81.00
Columbus OH	'CMH'	40.00	-82.89	40.00	-83.00
Cincinnati OH	'CVG'	39.05	-84.67	39.00	-84.50
Washington DC	'DCA'	38.85	-77.04	39.00	-77.00
Denver CO	'DEN'	39.86	-104.67	40.00	-104.50
Dallas TX	'DFW'	32.90	-97.04	33.00	-97.00
Detroit MI	'DTW'	42.21	-83.35	42.00	-83.50
Fresno CA	'FAT'	36.78	-119.72	37.00	-119.50
Grand Rapids MI	'GRR'	42.88	-85.52	43.00	-85.50
Honolulu HI	'HNL'	21.32	-157.92	21.50	-158.00
Westchester NY	'HPN'	41.07	-73.71	41.00	-74.00
Houston TX	'IAH'	29.98	-95.34	30.00	-95.50
Indianapolis IN	'IND'	39.72	-86.29	39.50	-86.50
Jacksonville FL	'JAX'	30.49	-81.69	30.50	-81.50
Las Vegas NV	'LAS'	36.08	-115.15	36.00	-115.00
Los Angeles CA	'LAX'	33.94	-118.41	34.00	-118.00
New York, NY	'LGA'	40.78	-73.87	41.00	-74.00
Kansas City MO	'MCI'	39.30	-94.71	39.50	-94.50
Orlando FL	'MCO'	28.43	-81.31	28.50	-81.50
Memphis TN	'MEM'	35.04	-89.98	35.00	-90.00
Miami FL	'MIA'	25.80	-80.29	26.00	-80.50
Milwaukee WI	'MKE'	42.95	-87.90	43.00	-88.00
Minneapolis MN	'MSP'	44.88	-93.22	45.00	-93.00
New Orleans LA	'MSY'	29.99	-90.26	30.00	-90.50

Mountain View CA	'NUQ'	37.42	-122.05	37.50	-122.00
Oklahoma City OK	'OKC'	35.39	-97.60	35.50	-97.50
Omaha NE	'OMA'	41.30	-95.89	41.50	-96.00
Chicago IL	'ORD'	41.98	-87.91	42.00	-88.00
Norfolk VA	'ORF'	36.89	-76.20	37.00	-76.50
Worcester MA	'ORH'	42.27	-71.87	42.50	-72.00
Portland OR	'PDX'	45.59	-122.60	45.50	-122.50
Philadelphia PA	'PHL'	39.87	-75.24	40.00	-75.00
Phoenix AZ	'PHX'	33.43	-112.01	33.50	-112.00
Pittsburgh PA	'PIT'	40.49	-80.23	40.50	-80.00
Providence RI	'PVD'	41.72	-71.43	41.50	-71.50
Winston-Salem NC	'RDU'	35.88	-78.79	36.00	-79.00
Richmond VA	'RIC'	37.51	-77.32	37.50	-77.50
Riverside CA	'RIV'	33.88	-117.26	34.00	-117.50
Rochester NY	'ROC'	43.12	-77.67	43.00	-77.50
Sacramento CA	'SAC'	38.51	-121.49	38.50	-121.50
San Diego CA	'SAN'	32.73	-117.19	32.50	-117.00
San Antonio TX	'SAT'	29.53	-98.47	29.50	-98.50
Louisville KY	'SDF'	38.17	-85.74	38.00	-85.50
Seattle WA	'SEA'	47.45	-122.31	47.50	-122.50
San Francisco CA	'SFO'	37.62	-122.38	37.50	-122.00
Salt Lake City UT	'SLC'	40.79	-111.98	41.00	-112.00
Saint Louis MO	'STL'	38.75	-90.37	38.50	-90.50
Tampa FL	'TPA'	27.98	-82.53	28.00	-82.50
Tulsa OK	'TUL'	36.20	-95.89	36.00	-96.00
Tucson AZ	'TUS'	32.12	-110.94	32.00	-111.00

FIGURES:

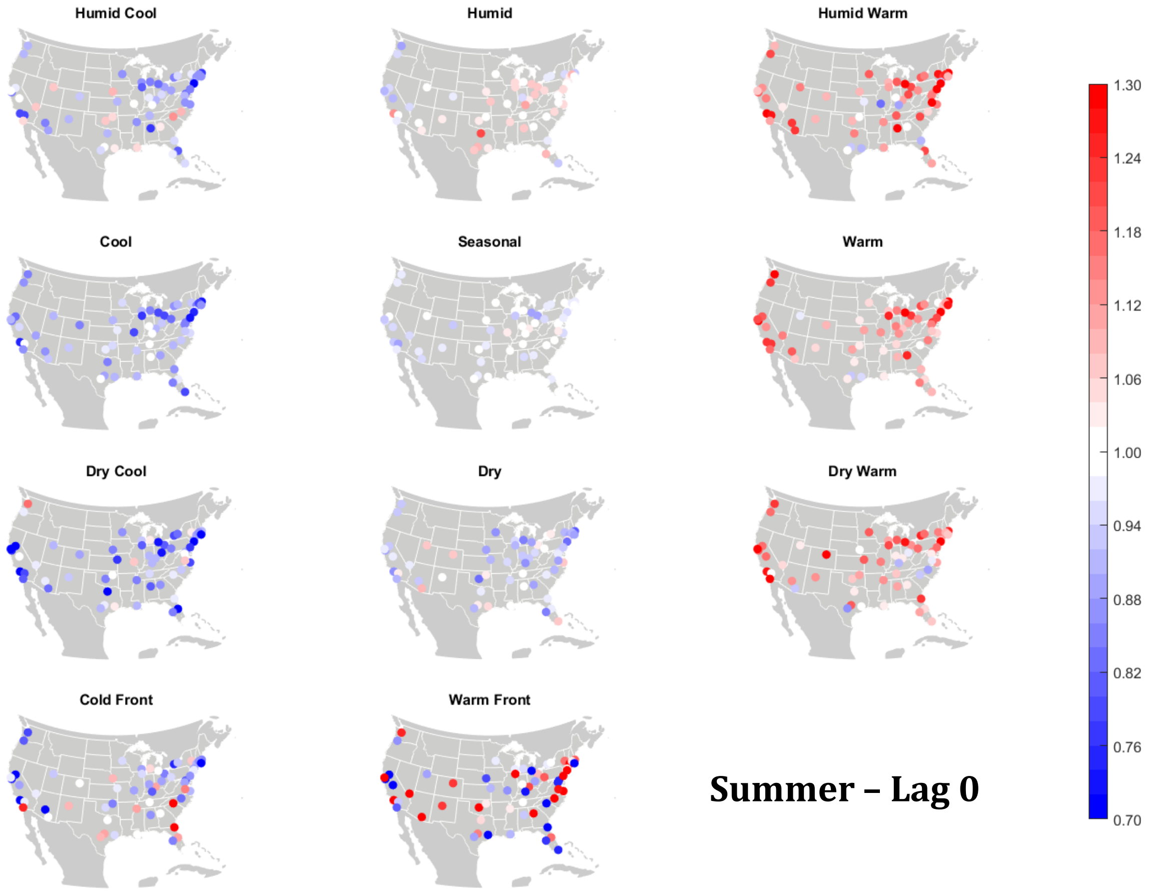

Figure S1: City-by-city relative risk of excess mortality in summer at Lag0 for each air mass.

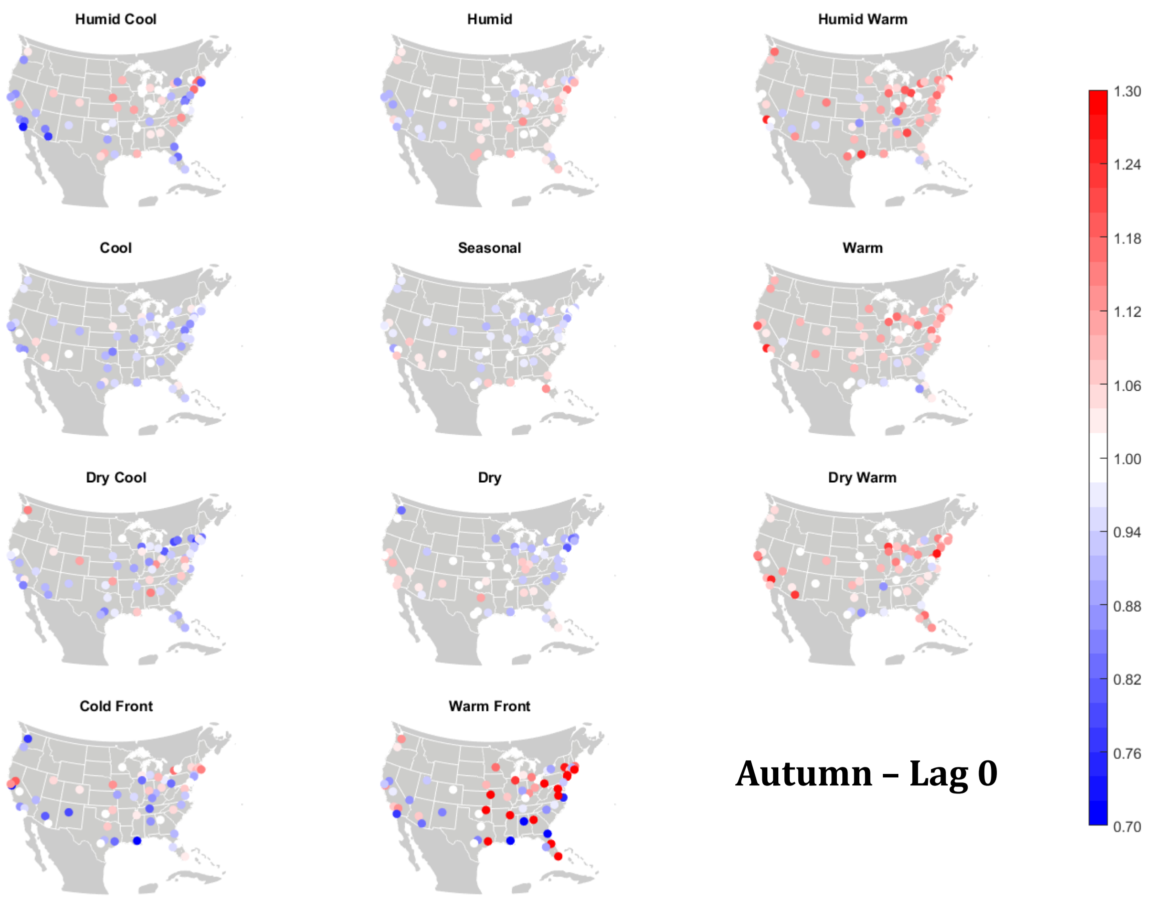

Figure S2: Same as Figure S1, but for autumn.

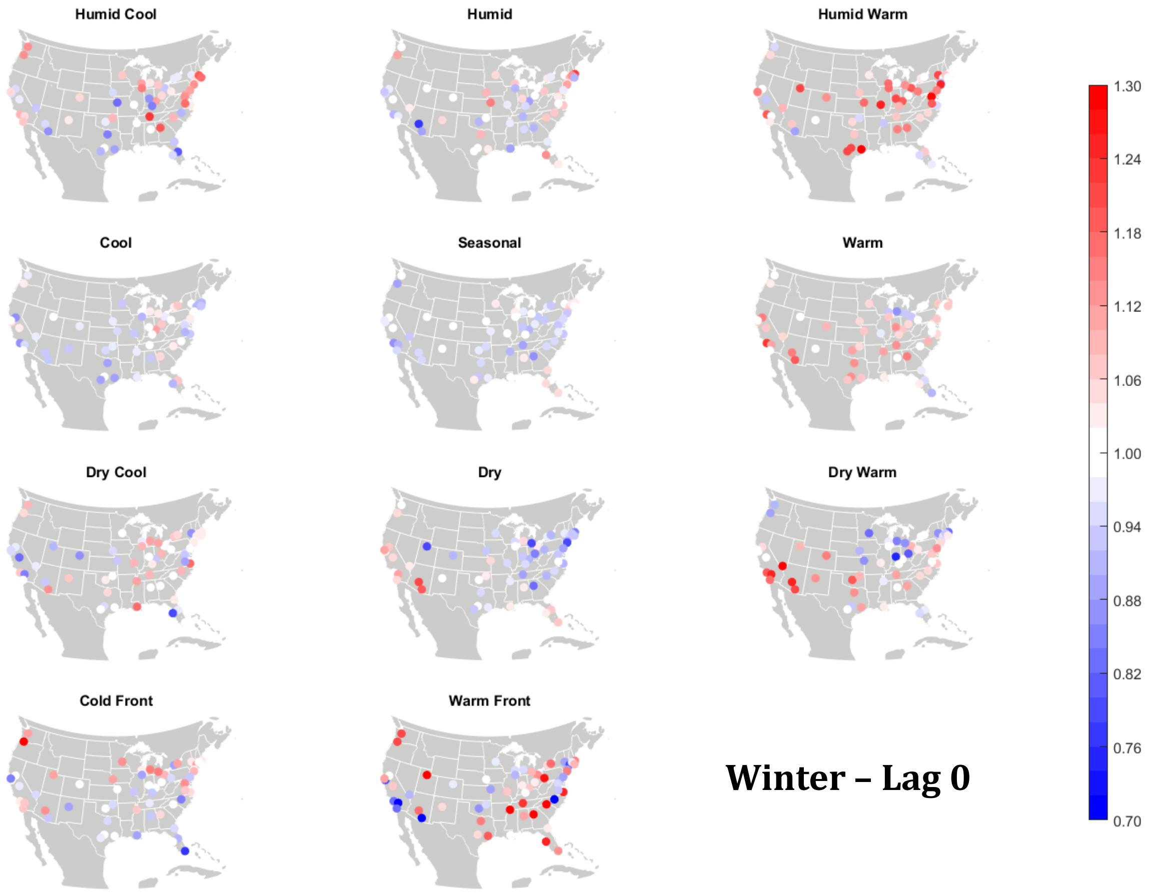

Figure S3: Same as Figure S1, but for winter.

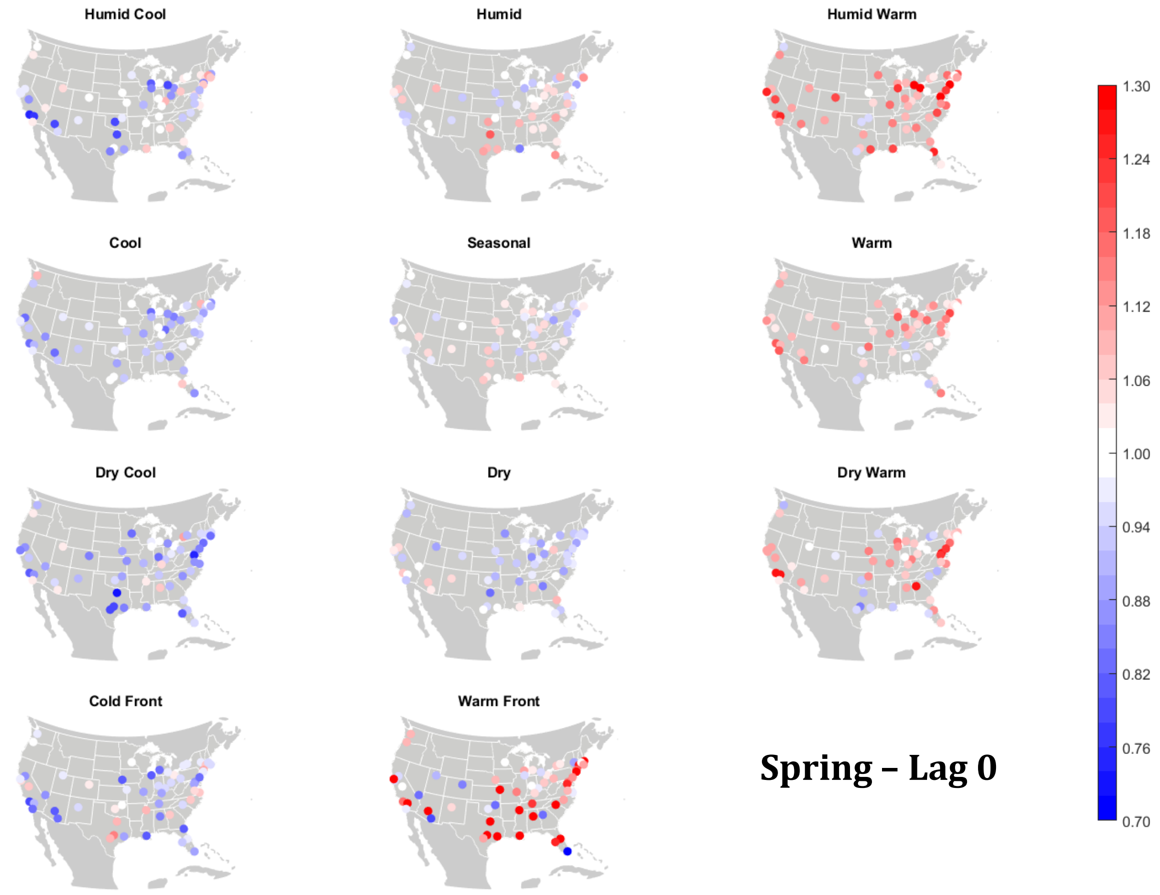

Figure S4: Same as Figure S1, but for spring.

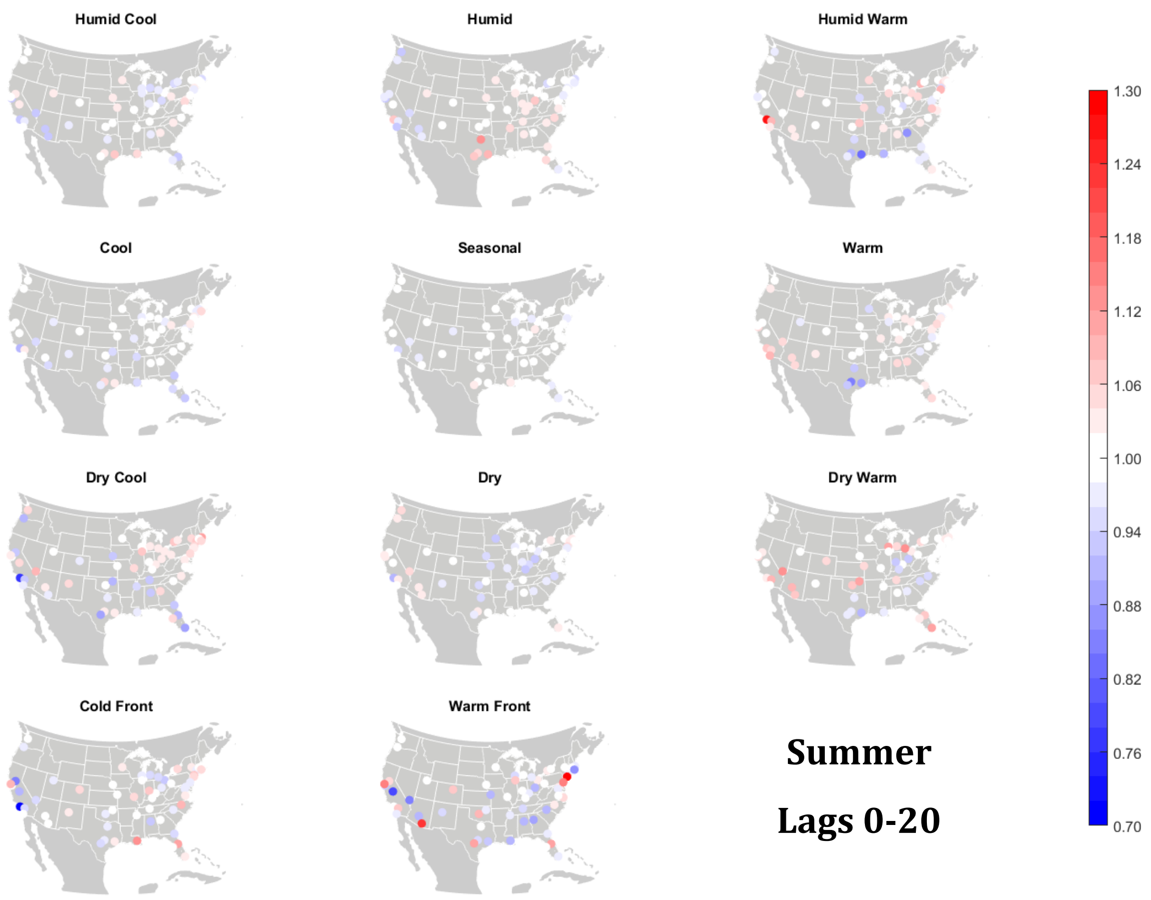

Figure S5: City-by-city relative risk of excess mortality in summer, averaged over Lags 0-20.

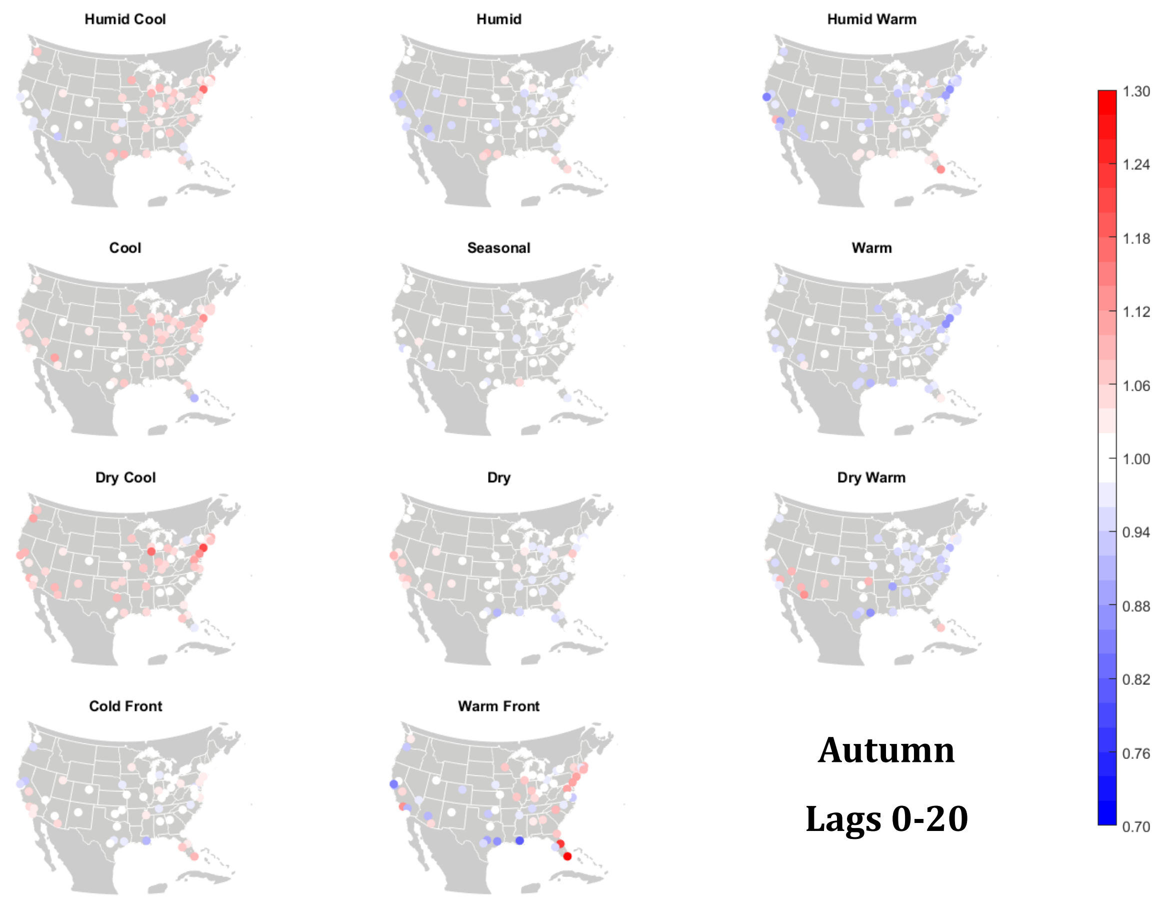

Figure S6: Same as Figure S5, but for autumn.

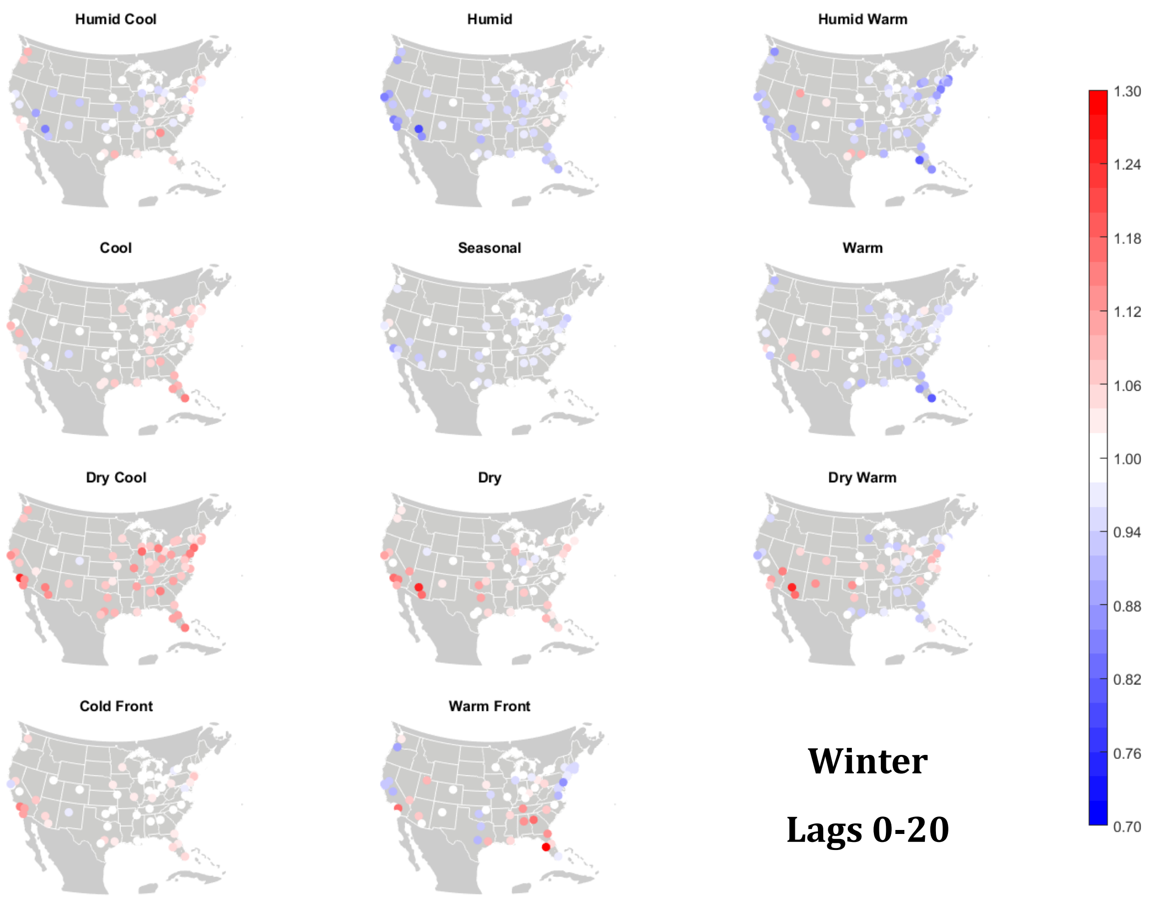

Figure S7: Same as Figure S5, but for winter.

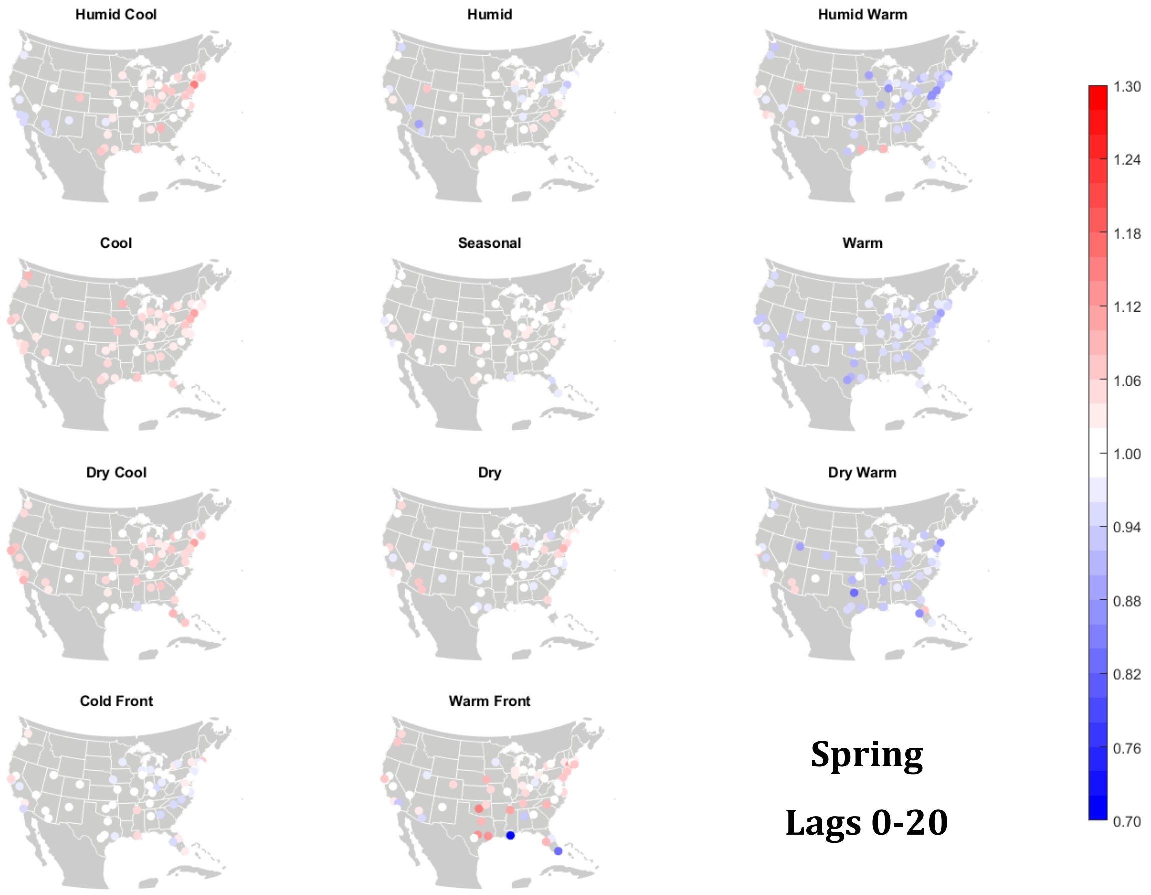

Figure S8: Same as Figure S5, but for spring.

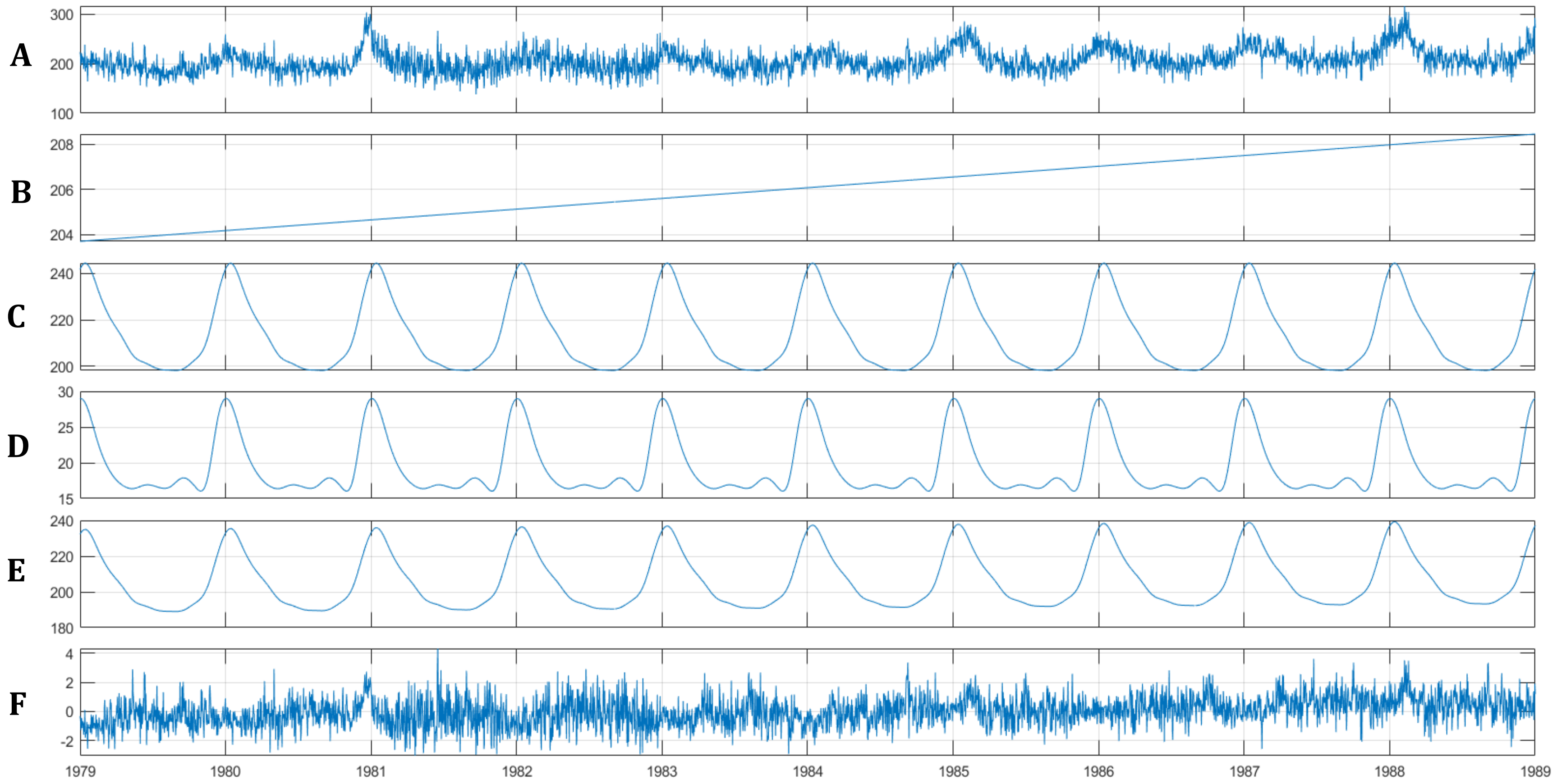

Figure S9: Example of detrending and deseasonalization process (for New York City (LGA) mortality data) for the first 10 years of the dataset. From top to bottom: A) raw data; B) linear trend; C) smoothed seasonal cycle of *mean* mortality; D) smoothed seasonal cycle of *standard deviation* of mortality; E) the combined linear trend and smoothed seasonal cycle of mortality means (i.e. the baseline) from which z-scores are calculated; F) the final analyzed data, Zmort, computed by: F = (A-E)/D.
